# Evaluating New Compounds to Treat *Burkholderia pseudomallei* Infections

**DOI:** 10.3389/fcimb.2018.00210

**Published:** 2018-06-25

**Authors:** Brittany N. Ross, Julia N. Myers, Laura A. Muruato, Daniel Tapia, Alfredo G. Torres

**Affiliations:** ^1^Department of Microbiology and Immunology, University of Texas Medical Branch, Galveston, TX, United States; ^2^Institute for Translational Science, University of Texas Medical Branch, Galveston, TX, United States; ^3^Department of Pathology, University of Texas Medical Branch, Galveston, TX, United States

**Keywords:** *Burkholderia*, antibiotic resistance, persistence, pathogen box, treatment

## Abstract

*Burkholderia pseudomallei* is the causative agent of melioidosis, a disease that requires long-term treatment regimens with no assurance of bacterial clearance. Clinical isolates are intrinsically resistant to most antibiotics and in recent years, isolates have been collected that display resistance to frontline drugs. With the expanding global burden of *B. pseudomallei*, there is a need to identify new compounds or improve current treatments to reduce risk of relapse. Using the Pathogen Box generated by Medicines for Malaria Venture, we screened a library of 400 compounds for bacteriostatic or bactericidal activity against *B. pseudomallei* K96243 and identified seven compounds that exhibited inhibitory effects. New compounds found to have function against *B. pseudomallei* were auranofin, rifampicin, miltefosine, MMV688179, and MMV688271. An additional two compounds currently used to treat melioidosis, doxycycline and levofloxacin, were also identified in the screen. We determined that the minimal inhibitory concentrations (MIC) for levofloxacin, doxycycline, and MMV688271 were below 12 μg/ml for 5 strains of *B. pseudomallei*. To assess persister frequency, bacteria were exposed to 100x MIC of each compound. Auranofin, MMV688179, and MMV688271 reduced the bacterial population to an average of 4.53 × 10^−6^% compared to ceftazidime, which corresponds to 25.1% survival. Overall, our data demonstrates that auranofin, MMV688197, and MMV688271 have the potential to become repurposed drugs for treating melioidosis infections and the first evidence that alternative therapeutics can reduce *B. pseudomallei* persistence.

## Introduction

*Burkholderia pseudomallei* is the causative agent of melioidosis, a disease originally of importance in Southeast Asia and Northern Australia (Currie et al., 2000; Sarovich et al., 2012a,b; Hatcher et al., 2015). However, a recent report found that global distribution of the pathogen is severely underreported and estimated that annually, *B. pseudomallei* causes 165,000 human infections and 89,000 deaths worldwide (Limmathurotsakul et al., 2016). Aside from being classified as a Tier 1 Select Agent due to its bioavailability and high potential of aerosolization, *B. pseudomallei* is a multidrug-resistant pathogen that is susceptible to very few antibiotics (Sarovich et al., 2012a,b; Ahmad et al., 2013). Depending on the clinical manifestations of the disease, treatment for *B. pseudomallei* is usually biphasic, starting with 10–14 days of intravenous therapy, followed by weeks to months of oral eradication therapy. The most commonly administered intravenous drugs consist of ceftazidime with or without trimethoprim-sulfamethoxazole (TMP-SMX), amoxicillin–clavulanic acid, imipenem, and cefoperazone/sulbactam (Estes et al., 2010). The second phase of treatment consists of a minimum of 3 months of oral chloramphenicol, TMP-SMX, and doxycycline, or amoxicillin-clavulanic acid (Estes et al., 2010).

Treatment failure has been reported in some cases due to antibiotic resistance. Clinical reports show that all *B. pseudomallei* species possess resistance against many classes of antibiotics and some isolates are also resistant to front-line antibiotics, such as ceftazidime and TMP-SMX, making treatment options limited (Sadiq et al., 2016; Cummings and Slayden, 2017). In addition to antibiotic resistance and the requirement for an extensive treatment regimen, an additional hurdle is that infection relapse occurs in 13–23% of patients (Chaowagul et al., 1993; Currie et al., 2000; Maharjan et al., 2005; Suntornsut et al., 2016). Though speculation has been made that these repeat cases could be due to re-infection, a recent study found that 75% of these recurrent cases are due to relapse, while only 25% are due to re-infection (Maharjan et al., 2005).

The ability of *B. pseudomallei* to generate persistent populations is thought to be a major contributor to latent infections which can recrudesce when the immune system is compromised (Chaowagul et al., 1993). Bacterial persistence is well documented to be associated with chronic infections and infection relapse (Zhang, 2014; Byndloss and Tsolis, 2016). Persistence is a mechanism by which a portion of an antibiotic susceptible population enters a dormant-like state, rendering antibiotics ineffective. Many bacterial genes have been identified to play a role in persistence, however, very no compounds have been developed to target persister populations. Additionally, new drug discovery cost an average of 802 million U.S. dollars and requires approximately 10 years from start of development to use in the clinic, and implementing new compounds into current treatment regimens is complicated (Adams and Brantner, 2006). To reduce economic burden and advance the speed at which novel drugs can be tested, many groups are investigating the potential for repurposing Food and Drug Administration (FDA)-approved drugs. This method involves screening large panels of FDA-approved compounds for efficacy against off-label conditions, such as those associated with infectious diseases. Importantly, the diversity of compounds included in these panels allows for testing non-traditional treatments against a wide variety of organisms. Here, we took the drug repurposing approach to explore new treatment options for melioidosis and demonstrate efficacy with anti-rheumatic and anti-kinetoplastid compounds against *B. pseudomallei*.

## Materials and methods

### Ethics statement

All manipulations of *B*. *pseudomallei* were conducted in CDC/USDA-approved and registered biosafety level 3 (BSL3) facilities at the University of Texas Medical Branch (UTMB), and experiments with select agents were performed in accordance with BSL3 standard operating practices. The animal studies were carried out in strict accordance with the recommendations in the Guide for the Care and Use of Laboratory Animals of the National Institutes of Health. The protocol (IACUC #0503014D) was approved by the Animal Care and Use Committee of the UTMB.

### Bacterial strains and culture conditions

*B. pseudomallei* strain K96243 was obtained from BEI Resources (Manassas, VA, USA). *B. pseudomallei* 576 was obtained from the Defense Science and Technology Laboratory (DSTL, UK); *B. pseudomallei* NCTC13178 and NCTC13179 were obtained from the Health Protection Agency Centre for Infections (HPA), UK; and *B. pseudomallei* MX2013 was obtained from CDC, USA. For all experiments, bacterial strains were stored at −80°C. Prior to use, strains were streaked onto Luria-Bertani agar with 4% glycerol (LBG) and grown for 36–48 h prior to use. For liquid cultures, 3–5 colonies were inoculated into LBG broth and grown for 16 h at 37°C.

### Pathogen box

The Pathogen Box was obtained from Medicines for Malaria Venture (MMV, Geneva, Switzerland). The bacterial culture of *B. pseudomallei* K96243 was prepared as described above. Compounds were solubilized as directed by MMV. Briefly, compound plates were allowed to thaw at room temperature and 90 μl of DMSO (Sigma) was added to reach a concentration of 1 mM and further diluted with PBS to reach 100 μM. DMSO was also diluted with PBS and served as a negative control. Bacteria were adjusted to 5 × 10^5^ CFU per ml and treated with a final concentration of 2, 5, 10, or 50 μM of each compound or DMSO control (0.2–1%). Plates were incubated at 37°C. At 16 and 24 h post incubation, turbidity was checked visually and wells with little or no growth were recorded. The 2 μM concentration for each compound correlated to the following: doxycycline 888.88 μg/ml, MMV688179 806.74 μg/ml, MMV688271 806.74 μg/ml, levofloxacin 722.5 μg/ml, rifampicin 1,645.9 μg/ml, auranofin 1,357 μg/ml, and miltefosine 815.14 μg/ml. For the remainder of the experiments the concentration was expressed as μg/ml. The list of all compounds tested can be found at https://www.pathogenbox.org/about-pathogen-box/composition.

### Minimum inhibitory concentrations (MICS)

Compounds were serially diluted two-fold in PBS to generate a suitable range of doses for testing. Overnight cultures were grown and adjusted to 1 × 10^6^ CFU/ml. One-hundred μl of bacterial suspension was mixed with 100 μl of compound suspended in culture media to yield a final bacterial concentration of 5 × 10^5^ CFU/ml. *B. pseudomallei* can effectively grow in 5% DMSO, so 2% was the highest final concentration of DMSO utilized for the MIC assays. Plates were incubated at 37°C and examined for growth inhibition at 24 and 48 h. An initial study was conducted examining a range from 1 to 400 μg/ml, and then further adjusted and repeated for MIC determination. The MIC was calculated as the lowest concentration that visually inhibited bacterial growth. The experiments were performed in duplicate with 2 biological replicates.

### Kill curve

**Figure 1:**
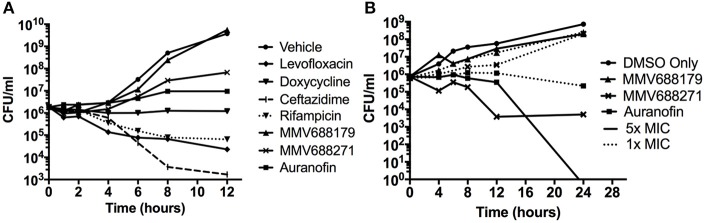
Characterization of Novel Melioidosis Treatment Compounds. **(A)** *B. pseudomallei* was cultured with 1x MIC at 37°C for 12 h. Over the course of time, CFUs were enumerated to determine if the compounds have a bacteriostatic or bactericidal activity. **(B)** *B. pseudomallei* was cultured with 1-5x MICs in the presence of 5% DMSO at 37°C for 24 h. A vehicle control was included to show the effect of 5% DMSO on growth.

**Figure 2:**
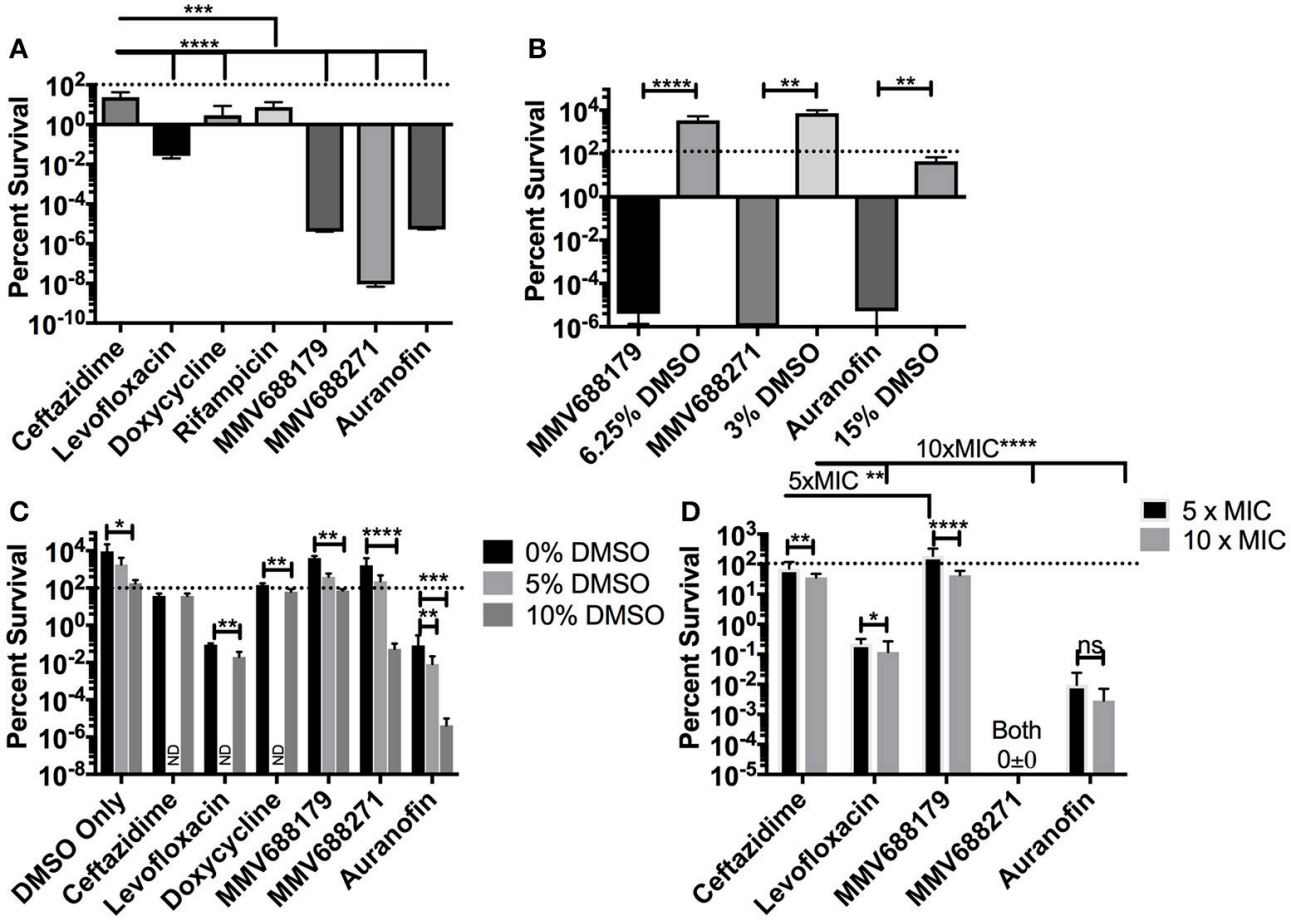
Determination of Persistence Frequency. **(A)** Persister frequency represented as percent survival of 1 × 10^8^ CFU/ml of *B. pseudomallei* exposed to 100x MIC of each compound. DMSO vehicle controls (15, 3, or 6.25%) and associated experimental conditions were tested in **(B)**. **(C)** To determine the effect of DMSO, persister assays were performed with 5x MIC of the compound and 0, 5, or 10% DMSO. Groups not investigated are labeled ND. All assays were grown at 37°C for 24 h and data presented as percent survival. **(D)** Persister assays were repeated at 5x MIC and 10x MIC to assess dose dependency on percent survival. Error bars indicate standard deviation and asterisks indicate a statistically significant difference between treatments (*P* < 0.05, ^*^P < 0.001, ^**^P < 0.001, ^***^P < 0.0001,^****^; ns, no statistically difference).

Overnight cultures were adjusted to 5 × 10^5^ CFU/ml and 1x MIC (Figure 1A) or 1–5x MICs (Figure 1B) of compound was added with or without 5% DMSO (Figure 2). Plates were incubated at 37°C and samples collected at 2, 4, 6, 8, 10, 12, and 24 h for CFU enumeration.

### Persister assay

To determining the persister frequency, bacteria were grown for 16 h and adjusted to 1 × 10^8^ CFU/ml in media containing 1, 5, 10, or 100x MIC in triplicate. After 24 h of incubation at 37°C, the surviving bacteria were assayed by serial diluting and plating on LB agar. Persistence was quantified by normalizing the surviving bacteria to the input concentrations and was expressed as percent survival. For each assay, a DMSO vehicle control only group was included to examine the potential effect of DMSO at the same concentrations used in the experimental groups. One-Way ANOVA with Kruskal-Wallis correction was used to determine if each compound had significantly different results compare to ceftazidime exposure (Figures 2A,C,D). *T*-test with a Mann-Whitney correction was used to compare each compound against the DMSO control (Figure 2B), and also to identify the significance of DMSO or dose effect for one compound (Figures 2C,D).

## Results

### MMV pathogen box

In this study, we used the MMV Pathogen Box to screen for compounds that inhibit *B. pseudomallei* K96243. Compounds were identified and further tested if they inhibited growth of the bacteria *in vitro*, as determined by reduced or lack of turbidity in comparison to vehicle-control-treated bacteria. To fully determine compound efficacy, we tested each compound at 2, 5, 10, and 50 μM/ml for growth inhibition. Of the 400 compounds tested, levofloxacin, doxycycline, auranofin, rifampicin, MMV688271, and MMV68817 were able to inhibit growth at all concentrations (Table 1). Miltefosine exposure resulted in consistent growth reduction and was further investigated to determine its MIC (Table 2). For all further studies, ceftazidime was used as a positive control since it is the most commonly recommended treatment for melioidosis (Estes et al., 2010).

**Table 1:** Identification of novel Melioidosis treatment compounds.

**Compound**	**Compound ID**	**Position**	**Trivial name**	**Total molecular weight**	**Molecular weight parent molecule**	**Molecular formula**
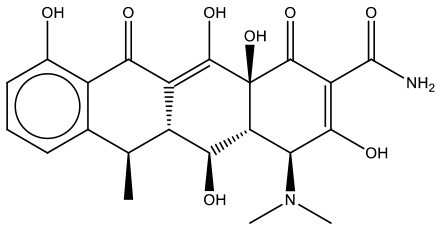	MMV000011	Plate B E04	Doxycycline	480.9	444.44	C_22_H_24_N_2_O_8_
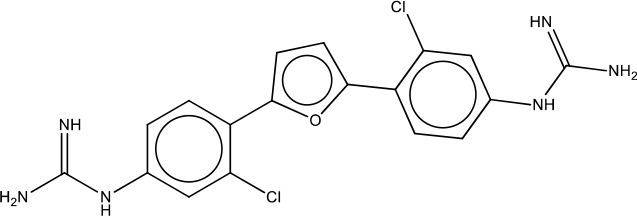	MMV688179	Plate C F03	NA	476.19	403.27	C_18_H_16_N_6_OCl_2_
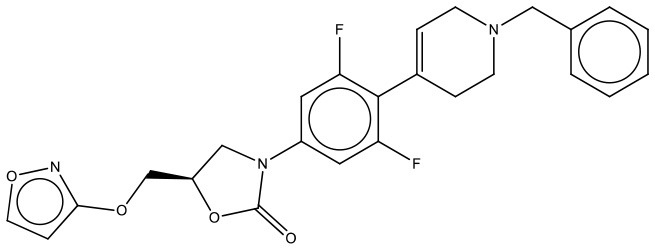	MMV688271	Plate D E10	NA	476.19	403.27	C_18_H_16_N_6_OCl_2_
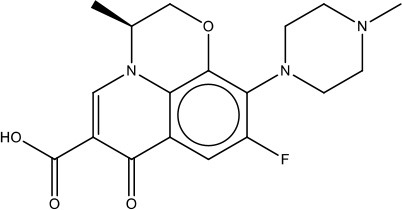	MMV687798	Plate E A05	Levofloxacin (-)-ofloxacin	361.37	361.37	C_18_H_20_N_3_O_4_F
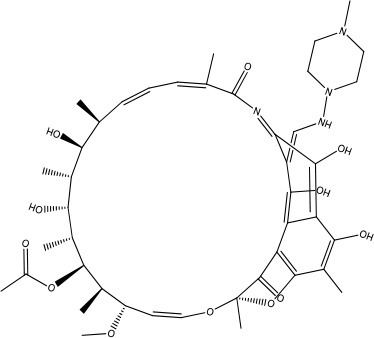	MMV688775	Plate E A06	Rifampicin	822.94	822.94	C_43_H_58_N_4_O_12_
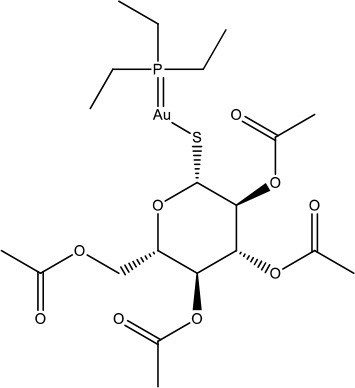	MMV688978	Plate E H05	Auranofin	678.48	678.48	C_20_H_34_AuO_9_PS
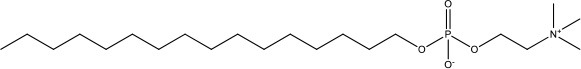	MMV688990	Plate E H06	Miltefosine	407.57	407.57	C_21_H_46_NO_4_P
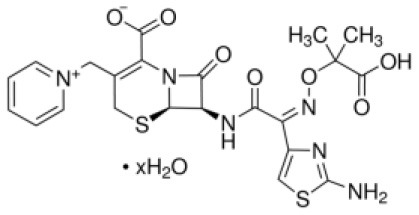	Added as control	NA	Ceftazidime	546.58	546.58	C_22_H_22_N_6_O_7_S_2_ · xH_2_O

**Table 2:** Minimal Inhibitory Concentrations.

**Minimal inhibitory concentrations**	**Levofloxacin (μg/ml)**	**Ceftazidime (μg/ml)**	**Doxycycline (μg/ml)**	**Auranofin (μg/ml)**	**Rifampicin (μg/ml)**	**Miltefosine (μg/ml)**	**MMV688271 (μg/ml)**	**MMV688179 (μg/ml)**
*B. pseudomallei* K96243	4	4	1	150	45	>1600	6	12.5
*B. pseudomallei* 576	10	4	2.5	150	18	>1600	12	>100
*B. pseudomallei* NCTC13178	6	4	3	150	18	>1600	10	>100
*B. pseudomallei* NCTC13179	6	6	2.5	150	25	>1600	8	>100
*B. pseudomallei* MX2013	6	3	2.5	150	18	>1600	12	>100

### Minimal inhibitory concentration (MIC)

As the first step in examining the efficacy of these compounds, we determined their minimum inhibitory concentrations (MIC) against 5 clinical isolate strains of *B. pseudomallei* from Australia, Thailand, or Mexico (Table 2). MMV688271, rifampicin, levofloxacin, and doxycycline were effective at concentrations below 50 μg/ml for all strains. The anti-kinetoplastid compound MMV688271 had an MIC ranging from 6 to 12 μg/ml. Rifampicin exhibited MICs below 50 μg/ml with a wide range (18–45 μg/ml) for the 5 strains, and was generally higher as compared to other compounds (e.g., ceftazidime). As expected, doxycycline, levofloxacin, and ceftazidime exhibited consistently low MICs and all have been previously used to treat patients with melioidosis (Table 2).

On the other hand, MMV688179, auranofin, and miltefosin had MIC ≥ 50 μg/ml. The MMV688179 compound exhibited an MIC of 12.5 μg/ml for *B. pseudomallei* K96243, although it was unable to inhibit growth up to 100 μg/ml for the remaining 4 stains. Due to limited availability of MMV688179, we did not evaluate higher concentrations. Auranofin exhibited a consistent MIC of 150 μg/ml for all strains. In our initial screen, miltefosine reduced the turbidity of the bacterial culture; however, when the concentration was increased to 1,600 μg/ml, miltefosine still could not fully inhibit bacterial growth and, therefore, was not further investigated in these studies.

### Kill curves

To determine whether the identified compounds had bacteriostatic or bactericidal activity, we conducted kill curves by treating bacterial cultures with low doses of the compounds normalized by their MIC. When treated with 1x MIC for 12 h (Figure 1A), we found that levofloxacin, ceftazidime, and rifampicin had bactericidal properties that began as early as 2 h and continued steadily until approximately 8 h post-treatment. At 8 h, levofloxacin, ceftazidime, and rifampicin treatment reduced the number of bacteria to 4.58 ± 6.48%, 0.26 ± 0.11%, and 5.58 ± 5.19%, respectively. Consistent with the literature, doxycycline exhibited bacteriostatic properties.

Auranofin treatment at 1x MIC inhibited growth in a bacteriostatic manner, while both anti-kinetoplastids were unable to inhibit growth. Because the anti-kinetoplastids and auranofin are DMSO-soluble, we also assayed for bacteriostatic or bactericidal properties in the presence of 5% DMSO (Figure 1B). As a control, *B. pseudomallei* was treated with 5% DMSO alone and we observed that the bacteria grew at three orders of magnitude above the input. With 5% DMSO and 1x MIC of auranofin combined, a bactericidal effect was observed and increased at 5x MICs, confirming its inhibitory properties. Both anti-kinetoplastids compounds did not show any effect with 5% DMSO and 1x MIC. However, when increased to 5x MICs, MMV688271 treatment had a bactericidal effect, reducing the mean bacterial concentration to 0.489 ± 0.68%, whereas MMV688179 did not show any effect. As a side note, we observed that MMV688179 requires more DMSO for full solubilization, which was corroborated by the visual presence of precipitated compound at 5% DMSO, and might be the reason of the lack of antibacterial activity.

### Persister assays

For many diseases, the generation of latent infection has been attributed to establishment of persister cell populations (Wood et al., 2013). It has been reported that *B. pseudomallei* K96243 has a persister frequency of 10^−1^ (percent survival of 10%) in the presence of 100x MIC of ceftazidime (400 μg/ml) (Butt et al., 2014). To test if our compounds of interest had an improved ability to inhibit the formation of persister cells compared to ceftazidime, we treated bacteria with 100x MIC for 24 h and assessed the persistence frequency by CFU enumeration normalized by input (Figure 2A). The results with levofloxacin and ceftazidime (both 400 μg/ml) are consistent with the literature (Butt et al., 2014), resulting in frequencies of 2.67 × 10^−2^ ± 6.99 × 10^−3^% and 23.87 ± 18.65%, respectively. Doxycycline (100 μg/ml) has a frequency of 2.911 ± 5.67%, while rifampicin, a drug not commonly used clinically due to high rates of resistance, had a persister frequency of 7.56 ± 7.563% (Figure 2A). Meanwhile, treatment with auranofin (1.5 mg/ml), MMV688179 (1,250 μg/ml), and MMV688271 (600 μg/ml) resulted in nearly complete eradication of the bacteria. In the few instances when bacteria was observed growing, only 1–4 CFUs were recovered on the 10^−1^ plate. The overall persister frequency and standard deviation for MMV688179 was 3.98 × 10^−6^ ± 7.89 × 10^−6^%, and for auranofin was 5.24 × 10^−6^ ± 1.48 × 10^−5^%. The MMV688271 compound inhibited bacterial survival to the greatest extent, with a persister frequency of 9.109 × 10^−9^ ± 2.25 × 10^−9^%. As indicated above, auranofin, MMV688179, and MMV688271 were solubilized with DMSO, which was carried over into the persister assays. To separately examine the effect of DMSO, vehicle controls with the equivalent concentration of the solvent alone were included (Figure 2B). The concentrations of DMSO at 3 and 6% contained bacteria counts that were higher than the input, showing that bacterial growth still occurred. At 15% DMSO, 54% bacteria remained, suggesting bacterial death. As expected, each control group had significantly higher bacterial counts compared to its associated treatment group, suggesting an effect of the compound on bacterial persistence and not just the DMSO (Figure 2B).

To further determine whether DMSO had an effect on compound efficacy, we exposed *B. pseudomallei* to 5x MIC of compound in the presence of 0, 5, or 10% DMSO (Figure 2C). For the vehicle control, only 10% DMSO significantly impacted growth of the bacteria but levels were not reduced below the bacterial input dose. At 0% DMSO, all compounds, except MMV688179, were significantly different (*P* < 0.03) from the DMSO only control. At 5% DMSO, only auranofin significantly reduced the bacteria count. Lastly, all compounds significantly (*P* ≤ 0.0001) reduced bacterial recovery compared to the 10% DMSO controls.

Lastly, to assess dose dependence on bacterial clearance, we adjusted each compound concentration to 5 and 10x MIC and enumerated the bacteria after 24 h (Figure 2D). At these concentrations, all drugs except MMV688271 showed a dose dependent decrease in efficacy between 5 and 10x MIC. When comparing all 5x MIC conditions, MMV688271 and auranofin showed improved killing compared to ceftazidime, while MMV688179 was less effective. The same was true when comparing all 10x MIC treatments vs. ceftazidime treatment.

## Discussion

*B. pseudomallei* is an important environmental bacterium that is able to cause severe infection and death if left untreated, and still causes a mortality rate of 40% when treatment is provided (Limmathurotsakul et al., 2016). With increasing global travel, *B. pseudomallei* can be introduced into previously non-endemic areas and persist in the environment (Limmathurotsakul et al., 2016). Today, in addition to Asia and Australia, the endemic presence of the pathogen has been confirmed in South America and the Caribbean. Of particular interest to the USA, reports indicate that Florida and certain regions of Texas are environmentally suitable for *B. pseudomallei*, further, increasing the threat of successful introduction into this country. Aside from the threat to North America, the increasing burden of this disease and isolation of antibiotic resistant strains could result in increased disease relapse, which is estimated to occur in 13–23% of cases (Limmathurotsakul et al., 2016).

This study aimed to identify and assess new treatment options or compounds that can complement existing melioidosis therapeutics. By screening the MMV Pathogen Box, we identified several compounds with different levels of efficacy against *B. pseudomallei*. Of the compounds identified, doxycycline is currently widely-used. Doxycycline is most efficacious when used for localized infections and for multi-drug treatment during systemic disease (Perumal Samy et al., 2017). Additionally, *B. pseudomallei* studies showed low rates of resistance to doxycycline when testing against 50 strains (2%) (Thibault et al., 2004). Levofloxacin is a promising compound that has been tested clinically due to its low MIC and high rate of bacterial killing. Here, we showed that of the available drugs, levofloxacin generated a smaller persister population than those reported for doxycycline and ceftazidime (Thibault et al., 2004; Estes et al., 2010; Butt et al., 2014). However, levofloxacin and other fluoroquinolones are generally not recommended for melioidosis due to high rates of relapse (Perumal Samy et al., 2017). Studies have also shown that among 50 *B. pseudomallei* isolates, 52% were resistant to levofloxacin, making treatment difficult unless new analogs with broader efficacy *in vivo* are generated (Thibault et al., 2004). Rifampicin also exhibits a moderate MIC and strong bactericidal activity against *B. pseudomallei*; however, like levofloxacin, resistance has been identified in 88% of the clinical isolates (Thibault et al., 2004).

The remaining three candidates (auranofin, MMV688179, and MMV688271) are novel agents with activity against *B. pseudomallei*. Although auranofin has a high MIC, all three were extremely effective at reducing persistent populations *in vitro*. This is particularly relevant, as persistent populations are important therapeutic targets due to their association with latent infection and relapse (Fauvart et al., 2011). Most importantly, these three drugs resulted in a significantly reduced frequency of persistent bacteria compared to all other compounds tested, including the antibiotics used in the clinic. This marks a milestone in therapeutic approaches in which novel compounds have been identified that can reduce *Burkholderia* persistent populations to 10^−8^–10^−10^ CFU/ml. Although the mechanisms of action against *B. pseudomallei* remain unclear, we have shown that auranofin and MMV688271 display bactericidal activity and are the best candidates for further detailed study.

Auranofin is a FDA approved compound that is used to treat rheumatoid arthritis and has been repurposed for other diseases such as HIV, cancer, parasitic, and bacterial infections (Harbut et al., 2015; Roder and Thomson, 2015; Thangamani et al., 2016; Wang et al., 2017). The known mechanism of action for auranofin is enzyme inhibition by irreversibly binding to thiol or selenol groups, resulting in the disruption of selenium metabolism and selenocysteine synthesis. Selenocysteine is required for the synthesis of glycine, proline, and thioredoxin reductases which are important for energy production (Asghari et al., 2017). In both *Clostridium difficile* and *Enterococcus faecalis* infection, auranofin has been shown to reduce selenium concentration and prevent production of selenoproteins, like in the case of glycine reductases (Jackson et al., 2006; Srivastava et al., 2011; Roder and Thomson, 2015). The major challenge moving forward with auranofin is its low bioavailability and high MIC. While generally administered orally, only 25% of the compound is absorbed and the peak concentration of 6–9 μg/ml is achieved after 20 min, which is far below the required MIC observed in this work (Roder and Thomson, 2015). In an attempt to increase availability, in a separate study we administered the compound to mice intraperitoneally at 10 mg/kg/day, which is just below the levels previously used in other treatments (Mirabelli et al., 1985; Ashino et al., 2011). We found that auranofin treatment alone did not provide significant protection against infection and further studies are needed to determine whether synergistic effects could be achieved when administered in combination with other compounds (data not shown). Alternatively, auranofin analogs are available and are being tested for other infectious diseases (Aguinagalde et al., 2015; Roder and Thomson, 2015). Such auranofin analogs offer the possibility of a lower MIC while maintaining inhibition of persister cell formation *in vitro*, which makes these compounds attractive candidates.

Both MMV688179 and MMV688271 are analogs to furamidine, which is an amphipathic diamine antiprotozoal drug. Pentamidine is as an aromatic diamidine used against human African *trypanosomiasis*. When Pentamidine's phenyl ring has been replaced with a furan ring, furamidine is generated and this drug is effective against human African trypanosomiasis. Furamidine in the form of a pro-drug is Pafuramidine, which is currently undergoing phase III clinical trials as a treatment for African trypanosomiasis (Ming et al., 2009; Pohlig et al., 2016). Furamidine has also shown efficacy against some Gram-positive cocci in wounds and burns, confirming their antibacterial potential and warranting the generation of analogs to test against a wide range of pathogens (Bichowsky-Slomnitzki, 1948). Although the mechanism of action of MMV688271 and MMV688179 remains unclear, it is known that these compounds interact with DNA at AT-rich sites and are believed to inhibit replication (Wang et al., 2000). Similar to Furamidine, analogs of these compounds have been shown to be effective against some Gram-positive cocci in wounds and burns, confirming their antibacterial potential (Bichowsky-Slomnitzki, 1948). We found that MMV688271 and MMV688179 can nearly eradicate *B. pseudomallei* at 100x MIC (600 and 1250 μg/ml, respectively). MMV688179 visibly precipitated at concentrations lower than 10% DMSO, making it a more unstable compound and potentially the reason for the lower efficacy. In contrast, MMV688271 proved to be more stable in solution and more effective at lower concentrations of DMSO.

Together, our data provides a strong rationale for further studies with the anti-kinetoplastid compounds, auranofin, or analogs that display improved solubility and lower MIC. Studies examining combination treatments with current antibiotics could be useful to generate novel persister eradication therapies. Future examination of such combinations and identifying synergistic or additive effects may be leveraged to improve current treatment plans. Overall, a drug repurposing approach for the testing of compounds against melioidosis showed that *B. pseudomallei* is resistant to many drugs compared to other bacteria tested against the same pathogen box; however, we successfully identified new compounds to be considered as anti-persister drugs. Importantly, this approach can be expanded to include additional platforms for compound discovery, in order to evaluate non-conventional therapies against *B. pseudomallei*.

## Author contributions

BR and AT designed research. BR performed the research with assistance by JM, DT, and LM, analyzed data, wrote the manuscript and was edited by JM, LM, and AT.

### Conflict of interest statement

The authors declare that the research was conducted in the absence of any commercial or financial relationships that could be construed as a potential conflict of interest.
